# Treatment of diabetic foot chronic ulcers and neuropathy with placenta-derived stem cells

**DOI:** 10.1093/stcltm/szag085

**Published:** 2026-11-03

**Authors:** Michael H Carstens, Arlen Beatriz Ríos, Juan Sevilla, Carlos Rivera, Osman Pravia, Dorian Calero, Samuel Vilchez, Kenneth A Bertram, Anthony Atala

**Affiliations:** Wake Forest Institute for Regenerative Medicine, Wake Forest School of Medicine, Winston-Salem, NC 27101, United States; Department of Surgery, Hospital Escuela Oscar Danilo Rosales Argüello, Universidad Nacional Autonoma de Nicaragua, León, Nicaragua; Department of Orthopedic Surgery, Hospital Escuela Cesar Amador Molina, Universidad Nacional Autónoma de Nicaragua, Matagalpa, Nicaragua; Department of Orthopedic Surgery, Hospital Escuela Cesar Amador Molina, Universidad Nacional Autónoma de Nicaragua, Matagalpa, Nicaragua; Department of Orthopedic Surgery, Hospital Escuela Cesar Amador Molina, Universidad Nacional Autónoma de Nicaragua, Matagalpa, Nicaragua; Department of Radiology, Hospital Escuela Cesar Amador Molina, Universidad Nacional Autónoma de Nicaragua, Matagalpa, Nicaragua; Department of Surgery, Hospital Escuela Oscar Danilo Rosales Argüello, Universidad Nacional Autonoma de Nicaragua, León, Nicaragua; Department of Radiology, Hospital Escuela Oscar Danilo Rosales Argüello, Universidad Nacional Autonoma de Nicaragua, León, Nicaragua; Department of Family Medicine, University of North Carolina, Chapel Hill, NC 27514, United States; Wake Forest Institute for Regenerative Medicine, Wake Forest School of Medicine, Winston-Salem, NC 27101, United States; Wake Forest Institute for Regenerative Medicine, Wake Forest School of Medicine, Winston-Salem, NC 27101, United States

**Keywords:** placenta stem cells, diabetic foot ulcer, diabetic peripheral neuropathy immunomodulation, angiogenesis

## Abstract

**Aim:**

This first in human study sought to determine the safety and clinical effects of placenta-derived stem cells (PSCs) administered by injection in a dose-escalation trial in diabetic subjects with foot ulceration (DFU) and peripheral neuropathy (DPN).

**Methods:**

Subjects were enrolled with non-healing wounds of 10-100 cm^2^ despite standard therapy for ≥3 months. Objectives included safety, wound healing, changes in neuropathy and in ankle/foot vascular flow. Ten patients received PSC implantation to the DFU circumference, into the ulcer bed and along the pedal arteries, with a total dose escalation of 30, 40, and 60 million (M) cells in 4, 3, and 3 subjects, respectively.

**Results:**

There were no PSC-treatment related serious adverse events. Wound healing was demonstrated in 10/10 subjects at 6 months. DPN improved in all subjects; with 9 of 10 achieving normal protective sensation by 12 months.

**Conclusions:**

Local implantation of PSCs for DFU and DPN is safe and of potential clinical value.

## Graphical Abstract

**Graphical Abstract:**
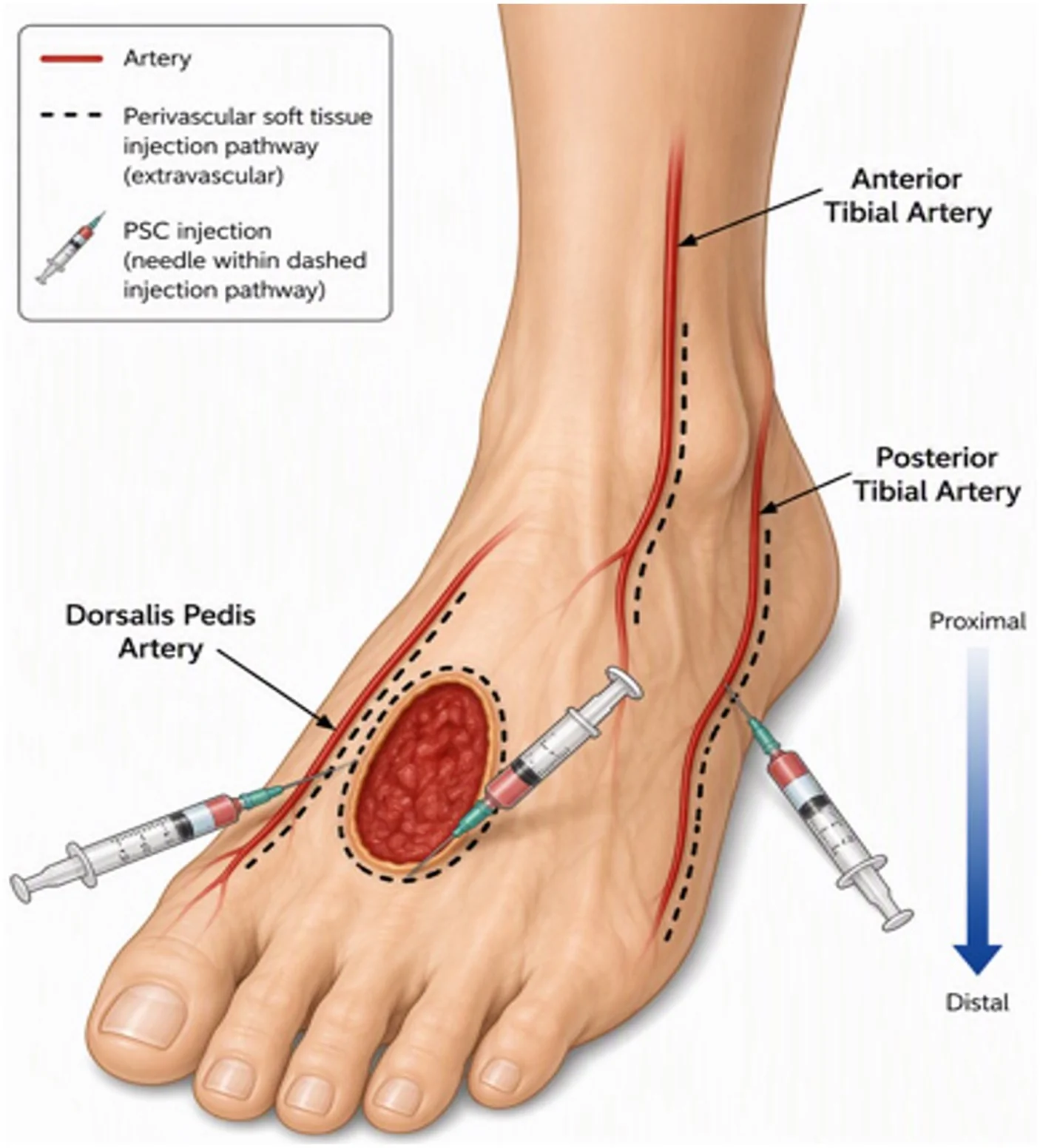

Significance statement Patients with diabetes mellitus frequently develop foot ulcers and neuropathyCell therapy offers the potential to heal nerve, vascular, and soft tissue injuries. Human allogeneic placental stem cells from normal deliveries were used in this small dose-escalation trial to treat diabetic foot ulcer and associated neuropathy. Soft tissue injection doses between 30 and 60 million cells were well tolerated without serious adverse events or dose-limiting toxicity. Wound healing was observed as early as 2 months after treatment. Exploratory studies demonstrated improved DPN in all subjects. Placental stem cells showed a favorable safety profile and potential clinical benefits.

## Introduction

Diabetes mellitus (DM) is a global public health threat, affecting 578 million (M) people by 2030 and consuming 10% of global health care expenditures.^1^ Diabetic foot ulcers (DFU) occur in 25% of all DM patients and poses a burden for health care systems, especially in resource-poor regions, where surgical revascularization options are limited, and patients are faced with undesirable options, such as chronic pain, infection, or amputation.^2^^,^^3^ Thus, a worldwide need exists for alternative DFU therapies.

DFU is closely associated with diabetic peripheral neuropathy (DPN), a progressive disease characterized initially by loss of pedal sensation, predisposing failure to perceive traumatic injuries with consequent ulceration. DPN also results in painful neuropathy in 20%-30% of patients.^4^ Pharmaceutical approaches such as tricyclics antidepressants, anticonvulsants, serotonin reuptake inhibitors, and opioids have failed to achieve adequate pain relief in most cases. No Food and Drug Administration (FDA)-approved therapies exist with disease modifying activity.^5^ Thus, new strategies for DPN are needed.^6^

Several types of adult derived mesenchymal stem cells have been used for the treatment of DFUs with varied results.^7–9^ Mesenchymal stem cells can be derived from different tissue sources, such as bone marrow, umbilical cord or fat, and can be used in an autologous manner. The therapeutic effects are largely due to paracrine factor production and short-term cell–cell contact.^10^^,^^11^

Embryonic stem cells (ESCs), obtained from in-vitro generated embryos, and induced pluripotent stem cells (iPSCs), generated by introducing transcription factors, proteins or small molecules into terminally differentiated cells to induce reprogramming into a stem cell phenotype, are the only pluripotent stem cell types described to date. Pluripotent stem cells have the advantage of extensive proliferation and differentiating into a variety of different cell types representative of all 3 germ layers—ectoderm, endoderm and mesoderm. Both ESCs and iPSCs have the potential to form teratomas in vivo.^12–14^

Placenta and amniotic fluid stem cells be isolated throughout gestation. Gestational tissue is usually discarded after birth, making this a plentiful source for stem cell retrieval. The placenta and amniotic fluid can be a source for various types of stem cells, including mesenchymal stem cells, or a more unique type of highly multipotent stem cells. The highly multipotent stem cells derived from the placenta or amniotic fluid, like ESCs and iPSCs, but unlike adult stem cells, are clonal, able to form cells from all 3 germ layers (ectoderm, endoderm, and mesoderm), and proliferate readily, making them available in large quantities for therapy. However, unlike ESCs and iPSCs, they do not form tumors.^15–17^ These cells also possess potent immunomodulatory properties, such as production of anti-inflammatory factors as well as interacting with immune cells to modulate the immune response at much higher levels than adult stem cells.^18^^,^^19^ The properties of placental derived stem cells (PSCs) make them an attractive alternative for therapy.

The purpose of this first in human study was to investigate the feasibility of using PSCs to treat DFU. The aim was to determine PSC treatment and dose safety for unilateral DFU disease, while also exploring their effects on wound healing, peripheral neuropathy, and vascular flow.

## Methods

### Subjects

#### Patient selection

Patients were identified from community health centers and subsequently referred to the orthopedic service of 2 university hospital where they were screened for eligibility by study physicians. Ten subjects were selected. All had been previously hospitalized for their wounds and discharged. The wounds had remained open for at least 3 months at the time of study enrollment despite standard of care measures in Nicaragua which consisted of wet-to-dry dressings changes and encouragement to avoid weight bearing. Two subjects with tissue erosion with exposed tendons and ligaments were candidates for surgical amputation at the time of study enrollment. Subject characteristics are listed in Table 1 (Results).

**Table 1.** Subject characteristics; cell dose received and location; and wound healing.

Subject ID	Age	Sex	Cell dose × 10^6^	Wound cm^2^ at PSC treatment	Closure timeline (months)
**01**	60	F	30	14	2
**02**	74	F	30	30	3
**03**	45	M	30	40	2
**04**	56	F	30	16	3
**05**	65	M	40	100	6
**06**	50	M	40	15	4
**07**	58	F	40	100	4
**08**	48	F	60	48	4
**09**	40	F	60	30	5
**10**	70	M	60	20	6

Abbreviation: PSC, placenta-derived stem cell.

*Inclusion criteria* were: (1) age 30-75 years; (2) clinically stable diabetes; (3) unilateral wound surface area ≥10 cm^2^; (4) presence of non-healing wound for ≥3 months; and (5) capacity to understand and provide informed consent. Patients with stable peripheral arterial disease were eligible for enrollment. Patients with acute limb-threatening ischemia requiring urgent revascularization were not considered candidates for enrollment. Vascular status was assessed using color Doppler ultrasonography because ankle-brachial index and toe-brachial index measurements were not routinely available within the study environment.

*Exclusion criteria* were: (1) presence of a medical condition prohibitive of intervention; (2) smoking and/or substance abuse within 3 months of study enrollment; (3) inability to fulfill study requirements; (4) evidence of clinically active wound infection, including purulent drainage, progressive cellulitis, abscess formation, systemic signs of infection, or clinical findings requiring antibiotic treatment or surgical debridement.

### Study design

This was a prospective, interventional open-label treatment safety first in human study, with exploratory efficacy outcomes, involving the administration of PSCs to treat unilateral DFU on a dose-escalation schedule and administered via injection into the subcutaneous and underlying tissues. Exploratory measures of treatment efficacy included: wound healing; changes in DPN; and changes in distal arterial flow.

Ten patients received PSC implantation distributed to the ulcer bed, the ulcer circumference, and along the pedal arterial vessels (see Graphical Abstract), with a total safety dose escalation of 30 M cells (4 subjects), 40 M (3 subjects), and 60 M (3 subjects). Patients were followed with weekly phone calls for the first 3 weeks, and in-person visits at 1, 3, 6, and 12 months after therapy.

Placenta-derived stem cells were prepared from healthy placentas at the time of delivery through a Wake Forest School of Medicine Investigational Review Board approved protocol. The clinical study was approved by the Ministry of Health (MINSA) of Nicaragua, and they received the PSCs through a Material Transfer Agreement, exported with the approval of the US FDA. Nicaragua was selected as the setting for this first-in-human investigation because diabetic foot disease represents a major unmet healthcare challenge in low- and middle-income countries, where patients frequently present with larger, more advanced wounds and where lower-extremity amputation rates remain substantially higher than those typically reported in developed healthcare systems. The study built upon a longstanding academic collaboration and prior Ministry of Health-approved cell therapy programs focused on limb salvage in patients with diabetic foot disease and critical limb ischemia. The study was conducted within the National Health Care system, with patient contact at regional community health centers in the Matagalpa region and referral to the regional academic medical center, Hospital Escuela Cesar Amador Molina. All clinical procedures were in accordance with the ethical standards of the Committee on Human Experimentation and the Helsinki Declaration of 1975, as revised in 2000. The study was approved by the Ministry of Health Institutional Review Board [number DGD-NVSQ-197-02-22]. Informed consent (including data/images for publication) was obtained from all study participants. The trial was registered in clinicaltrials.gov [NCT06373809].

### Baseline studies

After informed consent, patients had a history and physical exam, their medications were recorded, their DFU measured and photographed, and baseline evaluations conducted for DPN. Two types of sensation representing 2 separate pathways were assessed in each patient—fine touch (anterior columns) and vibration (posterior columns).

#### Fine touch

Sensory perception threshold (SPT) reflects small nerve fiber integrity. As such, it provides a sensitive metric defining the degree of DPN SPT and is commonly measured with the Semmes-Weinstein monofilament method consisting of plastic fibers of decreasing diameters representing different degrees of force (grams) required to cause flexion of the fiber accompanied by subject perception of force, thus documenting the threshold of sensation,^20^ (Figure S1). Plantar innervation presents a gradient of increasing thresholds with the smallest at the hallux, followed by the metatarsal heads and the heel.^21^ On this basis, a simplified system of SPT measurement was used, using 3 successive measurements on the heel.^22^ A diminished protective sensation at the heel occurs with a target force of 4-8 g while loss of protective sensation is present at a target force of 10 g.

#### Vibration

Vibration perception threshold (VPT) is considered relevant in the diagnosis of DPN. Vibration perception threshold provides a measure of large nerve fiber integrity and was assessed using the Horwell neuroesthesiometer (Algeos, Liverpool, UK),^23^ (Figure S2). An electric current mode varying from 0 to 50 volts (v) is converted into vibration of 50 Hz and transmitted to the patient using a vibrating stylus and applied perpendicular to the skin at defined points. The sequential measurements of VPT were obtained at the calcaneus. Values were graded as normal (<15 v); or mild (15-20 v), moderate (20-25 v), or severe vibration perception dysfunction (>25 v).^24^

#### Vascular flow measurements

Color doppler ultrasonography was performed to evaluate the pedal circulation of the affected foot via ultrasound studies. Dorsalis pedis artery circulation was tested for peak systolic velocity (cm/sec) on the dorsum of the foot at the midpoint between the metatarsal heads and the ankle. The anterior tibial artery was similarly assessed immediately above the ankle, while the posterior tibial artery was assessed posterior to the medial malleolus. Results were recorded by a single staff radiologist at each center.

### Processing, delivery, and storage of PSCs

Placenta-derived stem cells were prepared from placentas obtained at the time of normal deliveries from healthy mothers. Donors are screened and evaluated in accordance with the eligibility requirements outlined in 21 CFR Part 1271, Subpart C—Donor Eligibility. The donor’s health history is collected, and the donor is tested using FDA-approved donor screening tests for relevant viral and bacterial communicable diseases or agents. Once received at Wake Forest Institute for Regenerative Medicine, the placenta tissue is thoroughly rinsed with an antibiotic wash solution, the remaining amniotic membrane is separated from the placenta, after which the tissue is rinsed with a disinfectant solution and is cut into pieces. The fetal layer tissue is ground into pieces less than 1 mm and then enzymatically digested to release the cells that were bound to the connective tissue. Cells are then placed in tissue culture flasks for expansion in selected media to support stem cell growth and proliferation. The expanded PSCs are harvested at passage 4, washed, and then resuspended in CryoStor freezing medium solution for cryopreservation as a master cell bank. The cells are maintained in liquid nitrogen in the vapor phase.

Safety tests include PCR tests for viral components of animal origin and human adventitious agents. Final product testing consists of bacterial and yeast/fungal sterility cultures; and endotoxin and mycoplasma assays. Identity tests consist of flow cytometric immunophenotype evaluations for CD73 (+), CD105 (+), SSEA4 (+), CD45 (−), and HLA-DR (−). Stability tests consist of karyotype for genetic stability and long-term storage stability with cell count, viability, sterility, endotoxin, morphology, growth kinetics, and immunophenotype.

Placenta-derived stem cells were expanded from the master cell bank to passage 6 and then frozen at 10 M cells per 1 mL vial. The PSC vials were exported with the approval of the US FDA in a temperature-monitored liquid nitrogen container with direct delivery to the National Center for Diagnosis and Reference (Centro Nacional de Diagnóstico y Referencia; CNDR) at the National Health Complex in Managua. The cells were maintained on liquid nitrogen with an active temperature alarm system. For each subject treatment, the PSCs were transported on dry ice from CNDR to the treatment site on the day of intervention.

#### Randomizing, dosing and administration of PSCs cells

Upon arrival at the clinical site, the package containing the PSCs was verified as to its identity. The required number of cell vials (at 10 M PSCs per vial) were warmed to room temperature in an on-site 37° water bath. Once thawed, the cells were suspended in 37° Lactated Ringer’s (LR) solution in the dose and volume described below. They were subsequently injected via an #18-gauge needle.

The intervention at the foot was carried out by suspending the PSCs in LR. Three sequential groups of subjects were treated via local injections with increasing dosages of PSCs: 30 M cells (4 subjects), 40 M cells (3 subjects), and 60 M cells (3 subjects). Administration to the DFU/ankle was performed at 4 injection sites: (1) 20 cc into the wound bed, (2) 20 cc subcutaneously around the perimeter of the wound, (3) 10 cc along the posterior tibial artery pathway at the ankle prior to entry into the plantar surface, and (4) 10 cc along the anterior tibial artery/dorsalis pedis artery pathway from the ankle to the dorsum of the foot (see Graphical Abstract).

#### Post-intervention safety assessment

Patients were observed overnight in the hospital with vital signs and clinical observation for any potential (serious) adverse events [(S)AE] with emphasis on allergic reaction, ie, fever, rash, itching. After hospital discharge, patients were followed for (S)AE by telephone weekly for 3 weeks, and then with in-person visits at 1-, 3-, 6-, and 12-months after treatment. (S)AEs were categorized and graded using the Common Terminology Criteria for Adverse Events Version 5.0.^25^

#### Dose escalation

This early-phase, dose-escalation study was designed to evaluate the safety and tolerability of PSC therapy in patients with chronic wounds. Because this was a first-in-human investigation, dose selection was driven primarily by the need to assess tolerability across escalating cell doses rather than by expectations regarding efficacy.

A sequential cohort design was employed using 3 predefined escalating dose levels (30, 40, and 60 × 10⁶ cells), consistent with a modified 3 + 3 framework commonly used in first-in-human cellular therapy trials. Cohort sizes were intentionally small, reflecting the exploratory nature of early-phase dose escalation. Dose escalation proceeded after review of safety data from the preceding cohort. Fixed dosing, rather than wound-size-adjusted dosing, was intentionally used to allow independent assessment of safety across defined cell quantities without confounding from baseline wound burden. This approach also ensures clear attribution of any adverse events to a specific dose level. Exploratory evaluation of wound closure was included as a secondary endpoint to assess preliminary biological activity.

#### Exploratory efficacy assessments

Patients were evaluated with in-person visits at 1-, 3-, 6-, and 12-months. Assessments were focused on history, physical exam and wound healing, neuropathy, and distal leg artery flow changes as described above in the Baseline Studies section.

#### Statistical analysis

Primary (safety) and Secondary (dose escalation) Objectives—the incidence of (S)AE were recorded and relationships between any (S)AE and the intervention were determined. (S)AE differences between doses were noted.

Exploratory Efficacy Objective—the patients were to serve as their own controls, ie, baseline status versus outcomes at 12 months with regards to: (1) wound healing; (2) degree of peripheral neuropathy; (3) foot and ankle arterial blood flow. Analysis of difference in peripheral neuropathy using the Wilcoxon Signed test was used to determine significance of change in vibration sense between baseline and 12 months post-PSC treatment.

## Results

Ten patients with wounds that remained open for at least 3 months despite standard of care treatment, ages 40-74 years, were enrolled in this study, as shown in Table 1.

### Safety

Administration of PSCs was well tolerated by all patients. Patients were followed in the hospital for 24 hours with monitoring for potential (S)AE related to the PSCs cell injections (anaphylactic reaction; skin rash; bleeding; or thrombus/embolism). No changes in BP, heart rate, or respiratory rate were documented. All subjects reported a sensation of swelling in the treated leg after the PSC injections with resolution by the next day. After discharge, patients were contacted by telephone weekly for 3 weeks to assess for (S)AE. No skin reactions, infections, or hematomas or SAEs were reported in the 12-month follow-up period.

### Dose and wound healing response

Ten subjects were treated across 3 dose cohorts (30 × 10⁶, *n* = 4; 40 × 10⁶, *n* = 3; 60 × 10⁶, *n* = 3). As noted above, no dose-limiting toxicities or safety concerns were observed at any dose level, and treatment was well tolerated across all cohorts. Complete wound closure was achieved as early as 2 months in 2 patients and in all 10 evaluable patients by 6 months. All subject-reported wound closures were confirmed at the next in-person follow-up visit. No patients showed new ulcers in the index foot. It is of note that subjects with exposed tendons in the wound bed achieved full-thickness closure with a free range of motion as shown in Figure 1.

**Figure 1.**
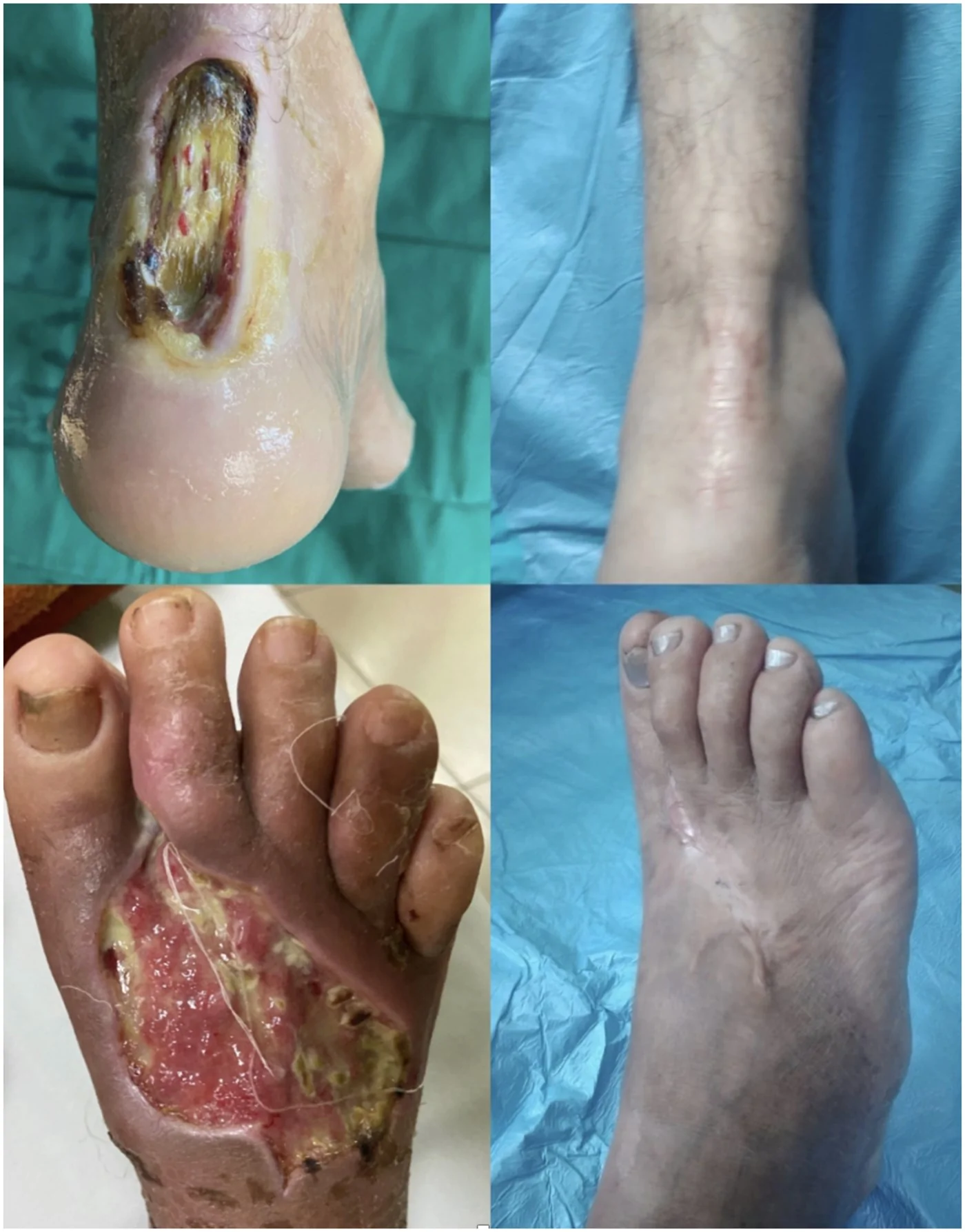
Healing of diabetic foot ulcers with exposed tendons. Upper left. 70-year old male with a left ankle supracalcaneal 21 cm^2^ ulcer and exposed Achilles tendon (baseline). Upper right. Soft tissue closure occurred at 6 months. Good tendon function with free range of motion. Lower left. 48-year old female with a 48 cm^2^ ulcer on the dorsum and exposed tendons (baseline). Lower right. Full closure at 4 months. At 12 months there was supple skin coverage with minimal contraction.

Mean wound closure times were 2.5 months in the 30 × 10⁶ cohort, 4.7 months in the 40 × 10⁶ cohort, and 5.0 months in the 60 × 10⁶ cohort. Baseline wound sizes varied among cohorts, with larger wounds represented in the intermediate dose group. Given the small number of patients per cohort and the variability in wound characteristics, closure outcomes were assessed descriptively, and the study was not powered to determine comparative efficacy between dose levels.

### Neurologic response

The baseline neuropathy evaluations (SPT and VPT) in the 10 subjects and respective changes over 12 months following PSC treatment are shown in Table 2. The transition of patients from significant SPT and VPT impairment categories at baseline to normal/protective SPT and VPT at 12 months is evident. A reduction in SPT protective sensation over the calcaneus is defined by a target force value of 4-8 g; complete loss of protective sensation over the calcaneus is defined by a target force value of ≥10 g. VPT values: normal (protective) (<15 v); or mild (15-20 v), moderate (20-25 v) or severe vibration perception dysfunction (>25 v).

**Table 2.** Changes in SPT and VPT scores baseline to 12 months after PSC treatment.

ID	Sensation (grams): Semmes-Weinstein				Vibration (volts): Neuroesthesiometer			
	Base-line	**3** **months**	**6** **months**	**12** **months**	Base-line	**3** **months**	**6** **months**	**12** **months**
**1**	10	10	2.0	0.4	20	10	4	1
**2**	300	0.4	0.4	0.4	20	12	10	1
**3**	300	2	0.4	0.4	20	10	10	1
**4**	300	0.4	0.4	4	20	15	9	3.5
**5**	300	ND	10	2	40	ND	45	4
**6**	300	10	4	2	40	11	20	8
**7**	300	0.4	0.4	0.4	35	11	10	2
**8**	10	4	4	0.4	30	15	10	5
**9**	2	0.4	0.4	0.4	20	15	10	10
**10**	300	4	300	2	50	40	25	3

Abbreviations: PSC, placenta-derived stem cell; SPT, sensation perception threshold; VPT, vibration perception threshold.

The 10 patients had a significant improvement in sensation (light touch) from baseline compared to 12 months post-PSC treatment as shown by Wilcoxon Signed Rank test: *P* = .005 (Figure 2). Furthermore, the patients demonstrated a significant improvement in vibration sense from baseline compared to 12 months post-PSC treatment as shown by Wilcoxon Signed Rank test: *P* = .005 (Figure 3).

**Figure 2.**
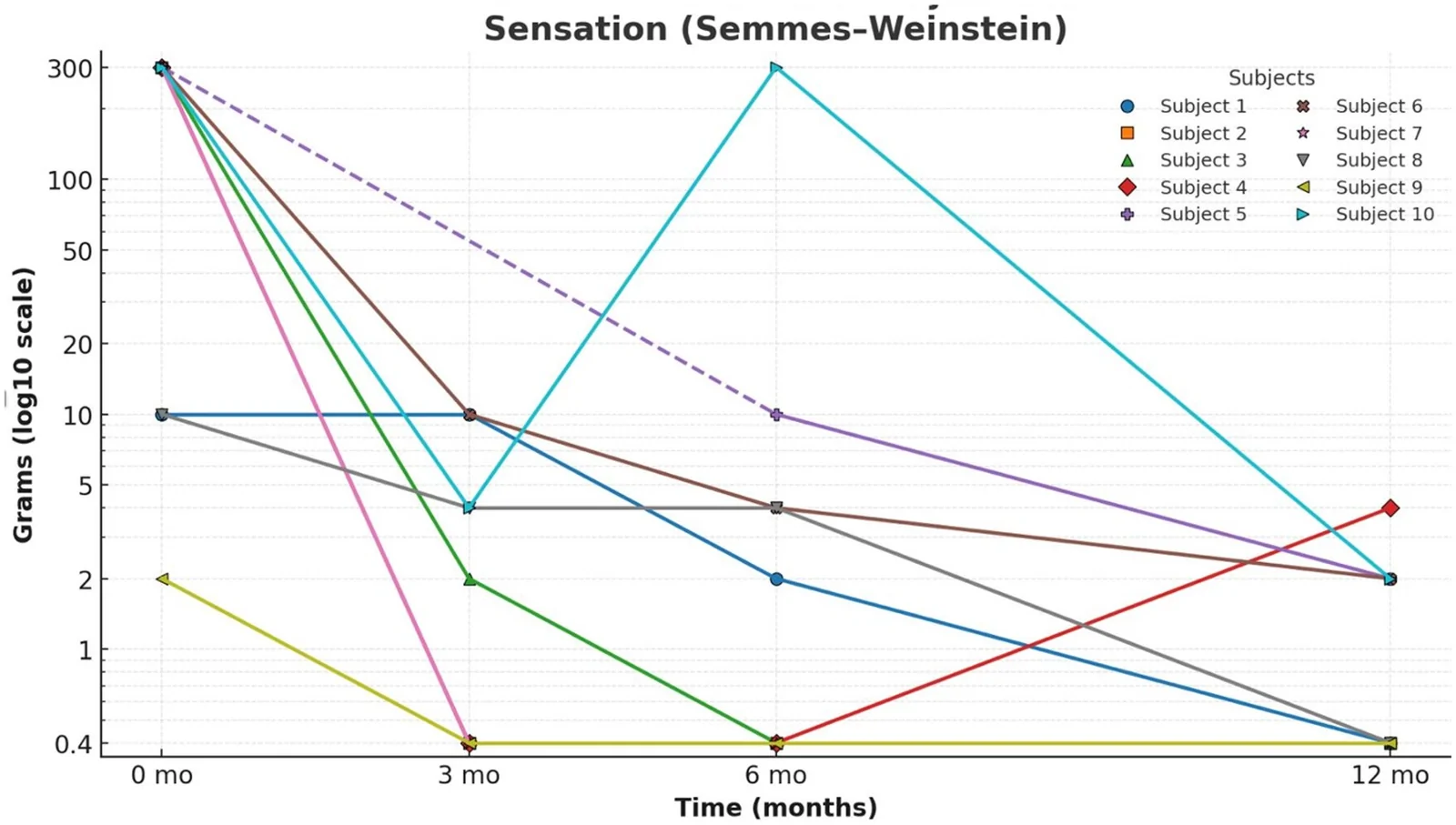
Changes in SPT over time: Subjects 1-10. Abbreviation: SPT, sensation perception threshold.

**Figure 3.**
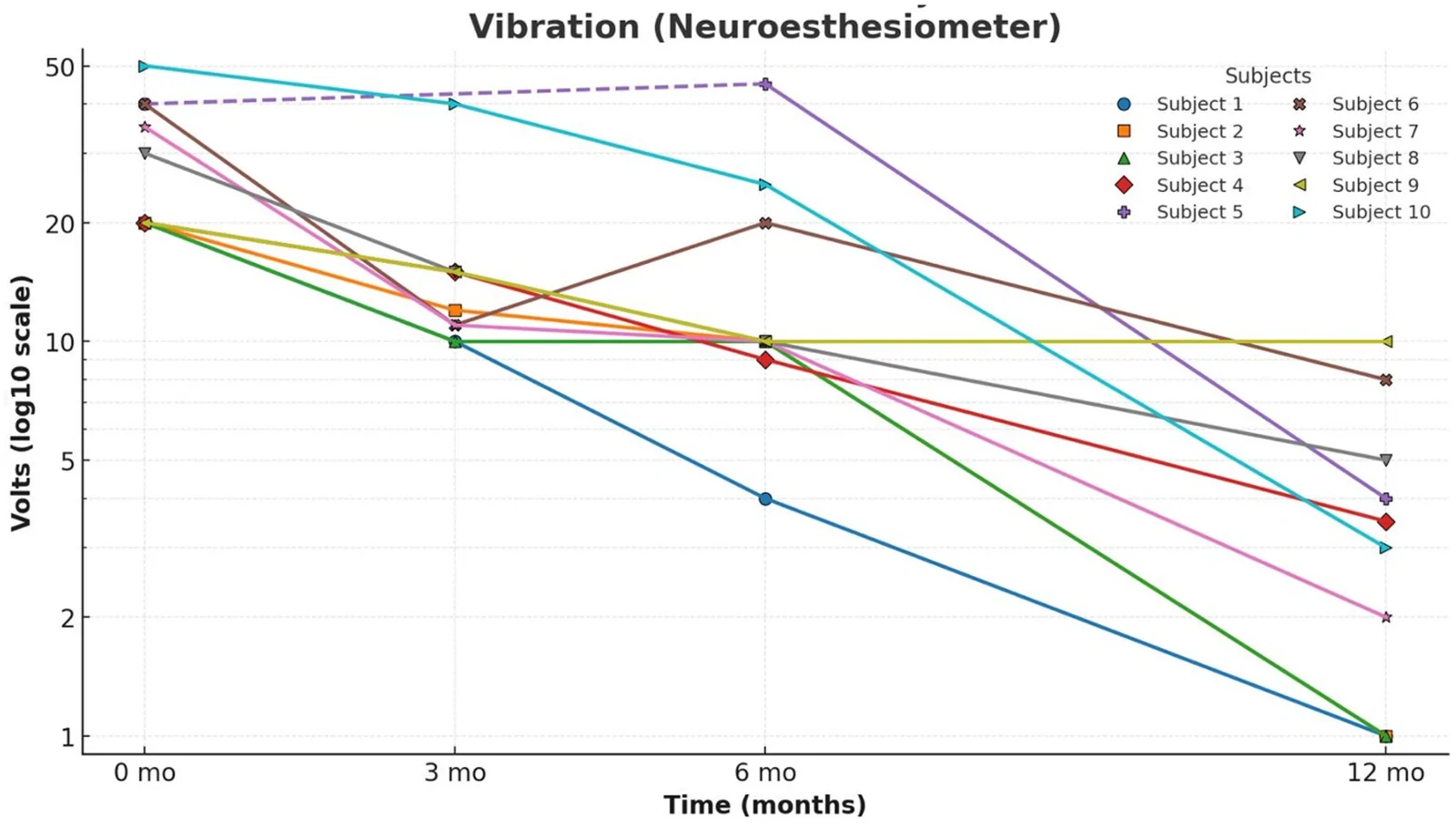
Changes in VPT over time: Subjects 1-10. Abbreviation: VPT, vibration perception threshold.

### Vascular response

Placenta-derived stem cells were implanted in 10 subjects along the course of 3 distal arteries in the foot, posterior tibial artery, anterior tibial artery, and dorsalis pedis artery. Ultrasound measurements of peak systolic velocity (cm/sec) at baseline and at 12 months are shown for the 3 distal arteries in the index leg in Table S1. Although 7 vessels improved from baseline “no flow” state to measurable flow at 12 months, changes in vascular flow over time were inconclusive.

## Discussion

This first-in-human study demonstrates that PSC therapy was well tolerated across the investigated dose range, with no dose-limiting toxicities or treatment-related serious adverse events observed during the study period. The absence of dose-limiting toxicities in all cohorts supports the tolerability of 30-60 × 10⁶ cells in this patient population.

All 10 subjects evaluable at 12 months had complete closure of their DFU wounds, ranging from 10 to 100 cm^2^ in diameter at enrollment. Closure was achieved as early as 2 months in 2 patients and in all 10 by 6 months. Exploratory wound healing observations indicate closure occurred across all doses, with timelines more closely associated with baseline wound size than with the size of administered PSC dose, suggesting that therapeutic activity may be achieved at the lower end of the tested range.

The use of fixed, non-wound size-adjusted dosing was appropriate for the primary safety-focused objective and 3 + 3 dose-escalation design. However, for subsequent studies aimed at evaluating efficacy and optimizing treatment response, dosing normalized to wound surface area is likely warranted. Per-cm² dosing would standardize biological exposure across patients and allow more interpretable assessment of dose–response relationships. Based on safety and exploratory activity in this study, the lowest tested dose (30 × 10⁶ cells) is a reasonable starting point for Phase II investigations.

Treatment of DPN has been primarily symptomatic in nature.^5^^,^^6^ The findings in this study support the ability of PSCs to functionally improve DPN in the index leg within 3 months of treatment and at doses as low as 30 M cells. There was no benefit to injecting more than 40 M cells. Improvements in DPN are important to help decrease the development and recurrence of DFU and reduce DPN-associated symptoms. The findings in this study suggest that PSCs may benefit diabetes-induced neuropathy, presenting a new opportunity for treatment. Although the primary objective of this first-in-human study was evaluation of treatment tolerability and dose escalation, the consistency of neurologic improvement observed across both sensory testing modalities suggests that DPN should be considered a prespecified efficacy endpoint in future controlled studies.

Placenta-derived stem cells which have been noted to be highly multipotent and can give rise to cells from all 3 germ layers that make up the human body, ectoderm, endoderm, and mesoderm, have many features that make them unique and well suited for off the shelf use in patients. The cells in this study used cultivation techniques that enhanced for the presence of highly multipotent stem cells. Placenta-derived stem cells also possess characteristics that support allogeneic administration. These cells demonstrate low immunogenicity through limited expression of major histocompatibility antigens, including HLA-DR, and through active modulation of host immune responses. Their ability to suppress inflammatory signaling and alter immune-cell activity may contribute to the absence of clinically apparent immune-mediated reactions observed in this study and supports their potential utility as an off-the-shelf regenerative cell therapy.

Alternatively, mesenchymal stem cells can also be isolated from the placenta; like mesenchymal cells from other sources, they have a mesodermal phenotype and are more limited in their differentiation and proliferative potential. Placental-derived mesenchymal cells have demonstrated a favorable safety profile in multiple clinical settings.^26–28^ Wu et al.^29^ previously demonstrated the safety of cultured placental derived mesenchymal stem cells in 15 DFU patients treated with doses ranging from 3 M up to 100 M injected into the index calf muscle twice, on days 1 and 8. Healing of DFU, 5 complete and 2 partial, was noted, although no DPN improvements were observed. Gibbons et al.^30^ studied the impact of intramuscular-injected cultured placental derived mesenchymal cells to treat DPN in 25 subjects in a phase 2 randomized, placebo-controlled study. While their study demonstrated some improvement in DPN, it was not statistically significant.

Concerns regarding long-term tumorigenicity and genomic instability are relevant to the development of any stem cell-based therapy. Unlike ESCs and iPSCs, PSCs have not been associated with teratoma formation. Furthermore, the PSC product used in this study underwent extensive characterization, sterility testing, phenotypic analysis, and genetic stability assessment prior to clinical use. No evidence of abnormal tissue proliferation, neoplasia, tumor formation, or treatment-related serious adverse events was observed during the 12-month follow-up period. Nevertheless, larger studies with longer follow-up will be required to more comprehensively evaluate long-term safety.

Wound healing is a series of coordinated interactions involving endothelial cells, fibroblasts, and inflammatory cells. Tissue repair requires angiogenesis, controlled inflammation, and remodeling, all dependent on cytokines and growth factors. Numerous animal studies have demonstrated that stromal/stem cells promote angiogenesis, modulate inflammation, and promote tissue remodeling.^31^ Placenta-derived stem cells have been shown to improve wound healing via immunomodulation through the increased production of interleukin-10; inhibition of the activation of the NF-kappa B pathway, and conversion of macrophages to the M2 (anti-inflammatory) state.^32^ In addition, they have been shown to induce angiogenesis and associated tissue repair via VEGF-production and other secreted factors to include insulin growth factors and interleukins.^33^ In a mouse model of DPN, the injection of human placenta-derived MSCs into the index lower leg led to location of the MSC along the damaged nerve. Compared to the control group, there was increased evidence of neurogenesis, enhanced Schwann cell activity, and improved myelin sheath structure, all resulting in an improved thermal stimulation response.^34^ Based on the observed concurrent improvement in wound healing and peripheral neuropathy, we hypothesize that PSCs function primarily through paracrine immunomodulatory and regenerative signaling mechanisms rather than direct cellular replacement. Together, these properties are the hypothesized mechanisms of action for the healing of DFU and improved light touch and vibration sensation demonstrated in this study.

All patients in this study showed wound healing and DPN improvement. A definitive peripheral vascular benefit was not demonstrated. Of 10 patients evaluable at 12 months, ultrasound determined blood flow velocities showed both increases and decreases over the study time. However, several patients who had no vascular flow at baseline showed flow 12 months after PSCs treatment. These results suggest that continued evaluation of PSCs for DFU, DPN, and peripheral vascular disease is warranted.

There were multiple limitations to this study. The small size of this study limits the safety evaluation of PSC for treating DFU, especially given the multiple DM-related potential complications. In addition, this study was uncontrolled and not blinded for the subjects or the study team and thus, any results for PSC–DFU (and DPN) efficacy are only hypothesis-generating. The addition of serum biomarkers for DFU/inflammation could have been helpful to establish the biologic correlates with DFU improvement. The same is true for impact on DPN. Also, bilateral testing for DPN status would have been helpful to understand a local versus systemic effect of the PSC injections. Future studies will be needed to increase the size of the study population, to include additional DFU classification systems, and a control, placebo arm to better demonstrate PSC safety and efficacy.

## Conclusions

No treatment-related serious adverse events were observed following PSC implantation in patients with DFU and DPN at doses ranging from 30 to 60 M cells. This study demonstrates that PSCs have DFU wound healing potential. A promising observation is the improvement of DPN across the study patients, which may be a potential new treatment option not only for DPN, but possibly other forms of peripheral neuropathy. Overall, the results support the need for continued investigation of PSCs to treat DFU and DPN.

## Supplementary Material

szag085_Supplementary_Data
