# Supplementary material for: Treatment of diabetic foot chronic ulcers and neuropathy with placenta-derived stem cells
Source: Stem Cells Transl Med. 2026 Nov 3;15(11):szag085. doi: 10.1093/stcltm/szag085 (PMC13730048; doi:10.1093/stcltm/szag085)
Supplement: Supplementary file 1 [file szag085_supplementary_data.zip › 2026-07-29 SCTM fig S2 vibrometer .pdf]

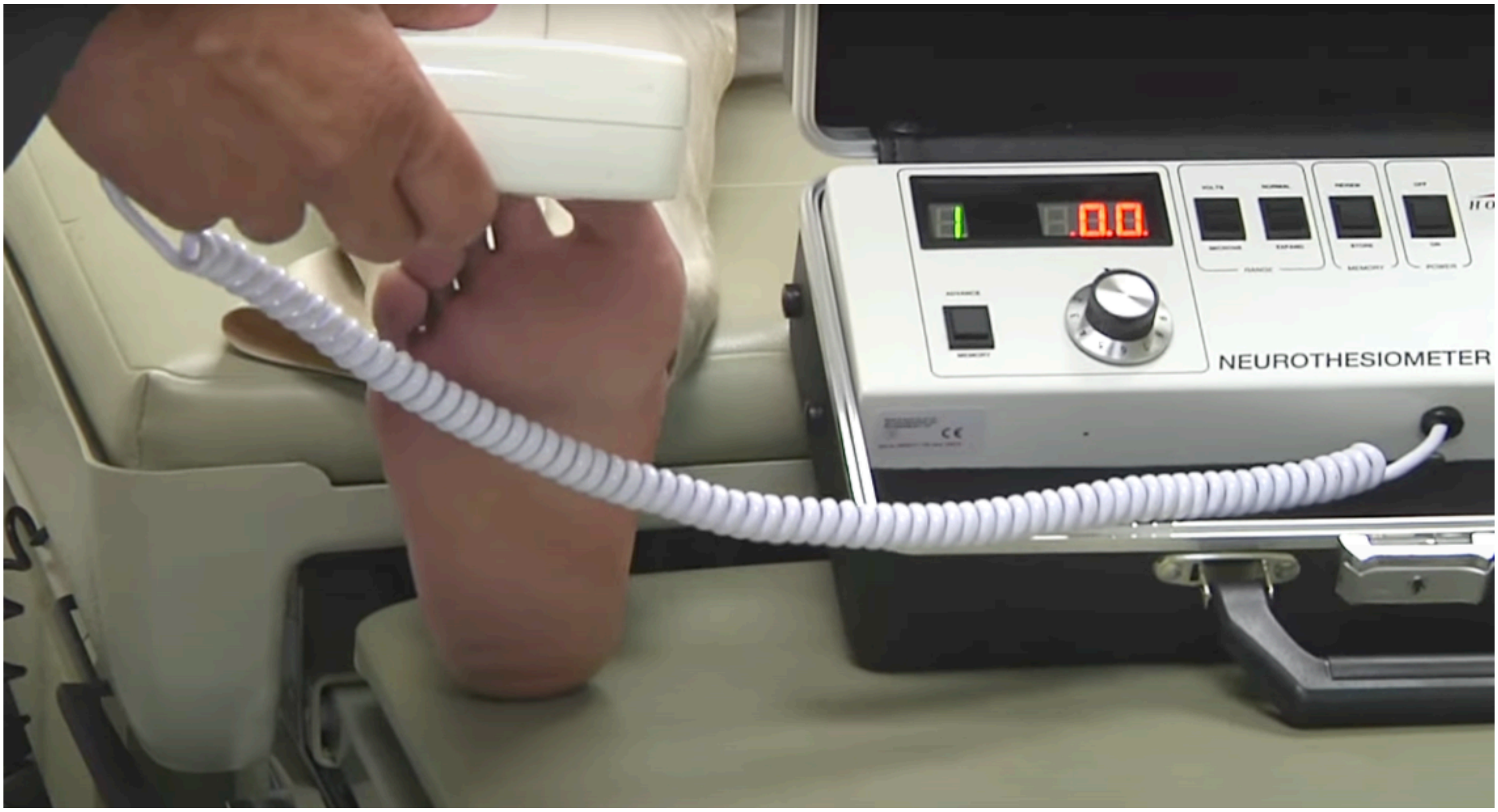
NEUROTHESIOMETER
