# Supplementary material for: Treatment of diabetic foot chronic ulcers and neuropathy with placenta-derived stem cells
Source: Stem Cells Transl Med. 2026 Nov 3;15(11):szag085. doi: 10.1093/stcltm/szag085 (PMC13730048; doi:10.1093/stcltm/szag085)
Supplement: Supplementary file 1 [file szag085_supplementary_data.zip › 2026-08-15 SCTM SUPPLEMENTARY INFORMATION - FINAL.docx]

**Treatment of Diabetic Foot Chronic Ulcers and Neuropathy with Placenta-Derived Stem Cells**

**Diabetic neuropathy: placenta stem cell treatment**

Michael H Carstens, MD^1-2^, Arlen Beatriz Ríos, MD^3^, Juan Sevilla, MD^3^, Carlos Rivera, MD^3^, Osman Pravia, MD^2^, Dorian Calero, MD^5^, Samuel Vilchez, MD, PhD^6^, Kenneth A Bertram, MD, PhD^1^, Anthony Atala, MD^#^

**Supplement: sensation assessment and pedal artery flow**

Figure S1. Sensory perception threshold testing using Semmes-Weinstein method

Legend. Semmes-Weinstein testing measured in milligrams required to detect flexion of nylon monofilament. Common scores reported for the calcaneus (point 9).

Figure S2. Vibration perception threshold testing using neuroesthesiometer

Legend. Neuroesthesiometer testing measured in volts required to detect vibration at 50 cycles per second. Common scores reported for the calcaneus (point 9).

Table S1. Distal pedal arteries – peak systolic velocity at baseline and at 12 months

| Subject  ID | **Posterior tibial artery** | | **Anterior tibial artery** | | **Dorsalis pedis artery** | |
| --- | --- | --- | --- | --- | --- | --- |
|  | T=baseline | T=12 mo | T=baseline | T=12 mo | T=baseline | T=12 mo |
| 1 | 27 | 36 | No Flow | 42 | 34 | 40 |
| 2 | 44 | 38 | 104 | 87 | No Flow | 43 |
| 3 | 97 | 50 | 75 | 50 | 21 | 82 |
| 4 | 54 | 80 | 84 | 76 | No Flow | 72 |
| 5 | 57 | 46 | 59 | 60 | No Flow | 72 |
| 6 | 44 | 114 | 45 | 91 | 44 | 92 |
| 7 | 76 | ND | 41 | ND | 118 | ND |
| 8 | No Flow | 64 | 44 | 60 | No Flow | 62 |
| 9 | 76 | ND | 42 | ND | 118 | ND |
| 10 | 26 | ND | 34 | ND | No flow | ND |

ND – test not done

Although seven vessels improved from baseline “no flow” state to measurable flow at 12 months, changes in vascular flow over time were inconclusive.
